# Chromatographic Analyses, In Vitro Biological Activities, and Cytotoxicity of *Cannabis sativa* L. Essential Oil: A Multidisciplinary Study

**DOI:** 10.3390/molecules23123266

**Published:** 2018-12-10

**Authors:** Gokhan Zengin, Luigi Menghini, Antonella Di Sotto, Romina Mancinelli, Francesca Sisto, Simone Carradori, Stefania Cesa, Caterina Fraschetti, Antonello Filippi, Letizia Angiolella, Marcello Locatelli, Luisa Mannina, Cinzia Ingallina, Valentina Puca, Marianna D’Antonio, Rossella Grande

**Affiliations:** 1 Department of Biology, Science Faculty, Selcuk University, 42130 Konya, Turkey; gokhanzengin@selcuk.edu.tr; 2 Department of Pharmacy, University “G. d’Annunzio” of Chieti-Pescara, 66100 Chieti, Italy; luigi.menghini@unich.it (L.M.); marcello.locatelli@unich.it (M.L.); rossella.grande@unich.it (R.G.); 3 Department of Physiology and Pharmacology “V. Erspamer”, Sapienza University of Rome, 00185 Rome, Italy; antonella.disotto@uniroma1.it; 4 Department of Anatomical, Histological, Forensic and Orthopedic Sciences, Sapienza University of Rome, 00185 Rome, Italy; romina.mancinelli@uniroma1.it; 5 Dipartimento di Scienze Biomediche, Chirurgiche ed Odontoiatriche, University of Milan, 20122 Milan, Italy; francesca.sisto@unimi.it; 6 Dipartimento di Chimica e Tecnologie del Farmaco, Sapienza Università di Roma, 00185 Rome, Italy; stefania.cesa@uniroma1.it (S.C.); caterina.fraschetti@uniroma1.it (C.F.); antonello.filippi@uniroma1.it (A.F.); luisa.mannina@uniroma1.it (L.M.); cinzia.ingallina@uniroma1.it (C.I.); 7 Department of Public Health and Infectious Diseases, Sapienza University of Rome, 00161 Rome, Italy; letizia.angiolella@uniroma1.it; 8 CeSI-MeT Centro Scienze dell’Invecchiamento e Medicina Traslazionale, Center of Aging Sciences and Translational Medicine, 66100 Chieti, Italy; valentina.puca@unich.it; 9 Department of Clinical Microbiology and Virology, Spirito Santo Hospital Pescara, 65124 Pescara, Italy; dantoniomarianna2@gmail.com

**Keywords:** *Cannabis sativa* L., chromatographic analysis, essential oil, antimicrobial activity, cancer cell cytotoxicity, naringenin, biofilm, antioxidant activity, *Galleria mellonella*

## Abstract

Due to renewed interest in the cultivation and production of Italian *Cannabis sativa* L., we proposed a multi-methodological approach to explore chemically and biologically both the essential oil and the aromatic water of this plant. We reported the chemical composition in terms of cannabinoid content, volatile component, phenolic and flavonoid pattern, and color characteristics. Then, we demonstrated the ethnopharmacological relevance of this plant cultivated in Italy as a source of antioxidant compounds toward a large panel of enzymes (pancreatic lipase, α-amylase, α-glucosidase, and cholinesterases) and selected clinically relevant, multidrug-sensible, and multidrug-resistant microbial strains (*Staphylococcus aureus*, *Helicobacter pylori*, *Candida*, and *Malassezia* spp.), evaluating the cytotoxic effects against normal and malignant cell lines. Preliminary in vivo cytotoxicity was also performed on *Galleria mellonella* larvae. The results corroborate the use of this natural product as a rich source of important biologically active molecules with particular emphasis on the role exerted by naringenin, one of the most important secondary metabolites.

## 1. Introduction

Hemp (*Cannabis sativa* L.) is often indicated as the oldest known, cultivated, and used plant by humanity. Its wide development was facilitated by broad soil and climate adaptation, allowing a large geographical distribution, as well as by crop flexibility, giving textile, fiber, food, feed, and biosolvents as final products or intermediates for manufacturing industries. A parallel history involves relative plants with secondary metabolism producing cannabinoids, and particularly tetrahydrocannabinoids (THCs), which characterize crops for medicinal or illegal recreational use due to psychotropic effects and addiction. As a consequence of the longtime debate on the correct taxonomic level of distinction between THC-producing or non-THC-producing plants, for most of the 20th century, a general prohibition of hemp cultivation existed. Currently, the non-THC-producing species of *Cannabis sativa*, or at least those producing a concentration lower than 0.2%, are usually referred to as fiber hemp, industrial hemp, and seed-oil hemp. Formerly, the maximum THC limit was imposed on plant cultivation in the open field and was not fixed by law with regards to final products such as food or nutraceuticals. The Italian Government promoted industrial hemp cultivation as a strategy to implement environmentally friendly crops and to contrast the loss of agricultural lands and green belts, as well as the desertification and the decrement of biodiversity [1].

In the Abruzzo region (central Italy), hemp cultivation is the object of renewed interest; both the surface dedicated to hemp crops and the volume of plant material produced are increasing. The economy of hemp crops is mainly sustained by female flower collection. This represents an innovative and high-value product that is used for tea infusion, to aromatize beer and other food products, and for nutraceutical purposes, involving extraction of important secondary metabolites [2]. Futura 75 cultivar was selected in France and is characterized by low THC content (<0.2%) and is widely cultivated for the production of fiber and seeds, while floral buds and leaves are considered as waste or by-products [3,4]. In this context, the essential oil (EO) obtained via hydro-distillation could represent a solution for the novelty and the multipurpose applications in medicinal, cosmetic, and food industries, because it could be obtained as primary product from female flowers or aerial parts, as well as from crop by-products, such as residual green parts (leaves and stems) after flower collection or from female inflorescences.

A previous study compared the composition and the antimicrobial activities of EOs obtained from three varieties of industrial hemp (Carmagnola, Fibranova, and Futura). Futura cultivar gave the highest essential oil yield and resulted in the most promising for application as an antimicrobial agent with a broad spectrum of activity [5]. Another comparative study was conducted on five hemp cultivars (Felina 34, Fedrina 74, SwissMix, Kompolti, and Secuemi) for chemical composition and antimicrobial activity. The most abundant compounds were found to be α-pinene, myrcene, *trans*-β-ocimene, γ-terpinolene, (*E*)-caryophyllene, and α-humulene. The disc diffusion test revealed moderate antimicrobial activity which different among the cultivars [6].

More recently, the EO obtained from inflorescences or leaves of hemp cv. Futura 75 was proposed as a potential source of botanical insecticides against larvae of *Spodoptera littoralis* and *Culex quinquefasciatus* and against adults of *Musca domestica* [4]. The essential oil was also tested in a field trial to control the rosy apple aphid pest and was determined as an ecological alternative to the acetamiprid [7]. An aqueous emulsion of the essential oil (0.02–0.1%), mainly characterized by (*E*)-caryophyllene (35%), β-myrcene (18%), and α-pinene (9%), induced high mortality rate when tested on the foxglove aphid (*Aulacorthum solani* Kalt.) and the two-spotted spider mite (*Tetranychus urticae* Koch) [8]. Hemp EO was also shown to fight the human-biting Asian tiger mosquito (*Aedes albopictus*) and the rice-crop invasive snail *Physella acuta* [9]. Increasing interest for the essential oil is confirmed by the increasing literature data, which could be related to its limited toxicity for mammals [10], as well as its low ecological impact on the environment [11].

Due to the increasing attention in the valorization of locally produced plants to support their use in the pharmaceutic, cosmetic, and food industry and to valorize the cultivation by-products, the EO obtained via hydro-distillation of aerial parts from *C. sativa* cv. Futura 75, cultivated in Italy, and its aromatic water were studied herein. Phytochemical investigation was performed on the terpenoid fraction (GC/MS), and the phenolic and flavonoid fingerprint (spectrophotometric and chromatographic methods), in addition to color analysis (CIELAB parameters; see Section 2.2). The potential applications of the EO were investigated in a wide multidisciplinary approach for different biological activities, such as antioxidant and antiradical, enzyme inhibition, antimicrobial activities on bacteria and fungi, and a comparative cytotoxic activity on tumor and non-tumor cell lines. A preliminary in vivo evaluation of the toxicity was also carried out on *Galleria mellonella* larvae. The broad-spectrum investigation provided a general characterization of the EO of THC-free *C. sativa* and could be used to support its use and application in innovative and multi-target ingredients for medicinal, cosmetic, veterinary, agronomic, or food use.

## 2. Materials and Methods

### 2.1. Experimental Farm and Plant Extraction

Two experimental fields were selected in the surroundings of Chieti, Italy (42°21′46.5′′ N 14°05′49.2′′ E, 100 m above sea level) in the old alluvial plain of the Pescara River Valley. The climate is Mediterranean, with an average yearly temperature around 15 °C and mean yearly rainfall around 750 mm. Summer can be variably dry. Total extension of the experimental crops comprises 15,000 square meters on level ground (slope 0–5%). During the two years preceding, the farm field was uncultured and mowed to obtain hay. The sowings were scheduled on the last week of March 2016 and were planned to favor the plant development during a long photoperiod and to stimulate full blooming in late summer.

The selection of Futura 75 was done considering the local climatic and soil characteristics in relation to the evidence of the high adaptive capabilities of this variety which are useful for multi-purpose cultivation. Selected seeds were purchased from South Hemp Tecno (Taranto, Italy) and certified by SOC France (Service officiel de contrôle et de certification des semences et plants) according to the plant variety database of the European Commission in the agricultural species list (position A-85, FR 8194). Soil tillage consisted of superficial ploughing followed by disc harrowing and final milling to prepare the sowing bed. Sowing was performed using a mechanic seeder for wheat, settled to obtain a final density of 50 kg of seeds/ha. The sowing was in line every 30 cm with 10 cm between lines. No fertilizer was used and, during growth, three irrigation cycles were programmed (automatic irrigation system, each cycle 24–36 h, depending on soil water availability). Plants development was monitored weekly until the beginning of flowering, when manual collection of aerial parts from flowering plants was done. The collection was carried out every three days, starting on the first week of September up to October 2016. Immediately after harvest, the plant material was transferred to the laboratory for extraction (about 100 m away). Random samples were tested for macroscopic characteristics in order to confirm their botanical identity (done by Prof. L. Menghini).

Fresh aerial parts consisting of leaves, inflorescences, and thinner residues of stem were roughly cropped to uniform size and accurately distributed inside the distillation chamber. The distillatory was a stainless-steel 12-L Clevenger-type apparatus (Albrigiluigi S.r.l., Stallavena, Italy). The distillation started within 5 h of plant collection in the field and was performed for no longer than 3 h. Straightaway, the collected EOs were passed through septa with anhydrous sodium sulfate in order to remove water residues, before being transferred to a sealed blue glass bottle and stored in the dark at 4 °C until used for phytochemical and biological assays. The EO yields (%, *v*/*w*) were determined each time and were expressed as mean volume obtained from plant fresh weight. Moreover, as a by-product, the aromatic water was also characterized in our experiments to assess its potential economic value in the framework of a lower-waste agri-food chain. In agreement with “circular economy” principles, our approach could further provide innovative results for reducing waste load.

### 2.2. Color Analysis

CIELAB parameters (L*, a*, b*, C*_ab_, and *h*_ab_), according to the “Commission Internationale de l′Eclairage”, were determined using a colorimeter X-Rite SP-62 (X-Rite Europe GmbH, Regensdorf, Switzerland), equipped with a D65 illuminant and an observer angle of 10°. Color interpretation was based on the lightness L* (between 0, absolute black, and 100, absolute white), the greenness (negative) or redness (positive) a*, and the blueness (negative) or yellowness (positive) b*. The chroma, or saturation (C*_ab_) quantitatively describes the color intensity, and the hue or color angle (*h*_ab_) is the attribute of appearance by which a color is identified according to its resemblance to red, yellow, green, blue, or a combination of two of these [12]. The results are reported as the mean value ± standard deviation (SD).

### 2.3. Gas Chromatography/Mass Spectrometry (GC/MS) Analysis

The volatile component analysis was carried out using an Agilent Technologies 6850 gas chromatograph (Santa Clara, CA, USA) coupled with an Agilent Technologies 5975 mass spectrometer (Santa Clara, CA, USA), equipped with an HP-5MS capillary column (5% phenyl 95% methylpolysiloxane, 30 m × 0.25 mm inner diameter, film thickness 0.25 µm; Hewlett-Packard, Palo Alto, CA, USA). GC parameters were adjusted as follows: injector temperature, 250 °C; flow rate of the helium carrier gas (99.995% purity), 1.0 mL/min. The oven temperature was set at 40 °C (5 min), then raised to 200 °C at 5 °C/min, and maintained at this temperature for 60 min. MS parameters were set as follows: energy of electron ionization, 70 eV; solvent delay, 6 min; source temperature, 230 °C; quadrupole temperature, 150 °C; and mass scan was carried out over the 50–350 *m*/*z* range.

The eluted compounds were identified by matching the relative mass spectra with those available from both a commercial database (FFNSC 3, Chromaleont srl, Messina, Italy) and online libraries (NIST 11, Flavor2, Scientific Instrument Services, Ringoes, NJ, USA) [13]. Kovats index (KI) was used as a second parameter to confirm the analyte identification. KIs were measured using a mixture of *n*-alkanes (C8–C24) in the same analytic conditions, and then compared with values reported in the literature and in the FFNSC 3 database. The identity of several compounds was confirmed through injection of standard samples available from commercial sources. The relative abundance of oil components was obtained by integrating the GC/MS peak areas without any further correction.

### 2.4. Total Phenolic Acid Content

The samples (1.0 mL) were added to 0.5 M hydrochloric acid (2 mL), 8.5% sodium hydroxide (2 mL), and Arnow reagent (10% aqueous solution of sodium nitrite and sodium molybdate, 2 mL), and diluted to 10.0 mL with water. The absorbance of the test solution was measured against the blank at 505 nm. Obtained results are reported as caffeic acid equivalents (CAE/g sample) [14].

### 2.5. Total Phenolic Content

The total phenolic quantification was determined using the Folin–Ciocâlteu method [15]. Test solutions (0.25 mL) were mixed with Folin–Ciocâlteu reagent (1 mL, 1:9 *v/v*). After 3 min, Na_2_CO_3_ solution (0.75 mL, 1%) was added and, after 2 h incubation at room temperature, the absorbance was measured at 760 nm. Results are expressed as milligrams of gallic acid equivalent per gram of sample (GAE/g sample).

### 2.6. Total Flavonoid Content

The total flavonoid quantification was measured using the AlCl_3_ method by Berk at al. (2011) [16]. Test solutions (1 mL) were mixed with aluminum trichloride (2% in methanol). Similarly, a blank was prepared (sample solution (1 mL) and methanol (1 mL)). The absorbances were recorded at 415 nm after a 10-min incubation at room temperature. Results are expressed as milligrams of rutin equivalent per gram of sample (RE/g sample) [17].

### 2.7. HPLC Analysis

All chemicals were purchased from Sigma-Aldrich (Milan, Italy), whereas HPLC-grade solvents were obtained from Carlo Erba Reagenti (Milan, Italy). Double-distilled water was obtained using a Millipore Milli-Q Plus water treatment system (Millipore Bedford Corp., Bedford, MA, USA). HPLC photo diode array (PDA) phenolic patterns were established using the validated method reported in the literature [18]. Data are reported as means ± standard deviation of three independent measurements.

## 3. Biological Activities

All biochemical analyses were carried out within four weeks of the extraction process to avoid any deterioration of the samples.

### 3.1. Radical Scavenging and Chelating Activity

#### 3.1.1. Free-Radical Scavenging Activity

Each test solution (1 mL) was added to 2,2-diphenyl-1-picrylhydrazyl (DPPH) solution (4 mL, 0.004% methanolic solution). The sample absorbance was noted at 517 nm after 30-min incubation at room temperature in the dark. Results are expressed as milligrams of trolox equivalent per gram of extract (TE/g sample) [19].

#### 3.1.2. Radical Cation Scavenging Activity

The 2,2-Azino-bis(3-ethylbenzothiazoline-6-sulfonic acid) (ABTS^+^) radical cation was produced in situ by reacting 7 mM ABTS solution with 2.45 mM potassium persulfate and allowing the mixture to stand for 12–16 h in the dark at the room temperature. Firstly, ABTS solution was diluted with methanol to an absorbance of 0.700 ± 0.02 at 734 nm. Each test solution (1 mL) was mixed with ABTS solution (2 mL). The sample absorbance was noted at 734 nm after 30-min incubation at room temperature. Results are expressed as milligrams of trolox equivalent per gram of extract (TE/g extract) [20].

#### 3.1.3. Evaluation of Total Antioxidant Capacity Using Phosphomolybdenum Assay

Each test solution (0.3 mL) was mixed with 3 mL of reagent solution (0.6 M sulfuric acid, 28 mM sodium phosphate, and 4 mM ammonium molybdate). The sample absorbance was read at 695 nm after 90 min incubation at 95 °C. Results are expressed as millimoles of trolox equivalent per gram of dry extract (TEs/g extract) [21].

#### 3.1.4. Cupric Ion Reducing (CUPRAC) Method

Each test solution (0.5 mL) was added to reaction mixture containing CuCl_2_ (1 mL, 10 mM), neocuproine (1 mL, 7.5 mM), and NH_4_Ac buffer (1 mL, 1 M, pH 7.0). Similarly, a blank was prepared using sample solution (0.5 mL) and reaction mixture (3 mL) without CuCl_2_. The absorbances were read at 450 nm after 30 min of incubation at room temperature. Results are expressed as milligrams of trolox equivalent per gram of extract (TE/g extract) [22].

#### 3.1.5. Ferric Reducing Antioxidant Power (FRAP) Method

Each sample solution (0.1 mL) was added to FRAP reagent (2 mL) containing acetate buffer (0.3 M, pH 3.6), 2,4,6-tris(2-pyridyl)-*s*-triazine (TPTZ) (10 mM) in 40 mM HCl, and ferric chloride (20 mM) in a ratio of 10:1:1 (*v*/*v*/*v*). Then, the absorbance was read at 593 nm after a 30-min incubation at room temperature. Results are expressed as milligrams of trolox equivalent per gram of extract (TE/g extract) [22].

#### 3.1.6. Metal Chelating Activity on Ferrous Ions

Each test solution (2 mL) was added to FeCl_2_ solution (0.05 mL, 2 mM). The reaction was initiated by the addition of 5 mM ferrozine (0.2 mL). Similarly, a blank was prepared using test solution (2 mL), FeCl_2_ solution (0.05 mL, 2 mM), and water (0.2 mL). Then, the absorbances of sample and blank were noted at 562 nm after 10-min incubation at room temperature. Results are expressed as milligrams of ethylenediaminetetraacetic acid (EDTA) equivalent per gram of extract (EDTAE/g extract) [22].

### 3.2. Antimicrobial Activity

#### 3.2.1. Staphylococcus aureus

##### 3.2.1.1. Bacterial Strains and Culture Conditions

The antimicrobial activity was evaluated on one reference strain (*S. aureus* American Type Culture Collection (ATCC) 29213) and three clinical strains (*S. aureus* 101 TV, *S. aureus* 104, and *S. aureus* 105), isolated from different sources and characterized by a different antimicrobial susceptibility pattern. The samples were cultured on Mueller–Hinton agar (MHA, Oxoid Ltd, Hampshire, UK) and on mannitol salt agar (MSA, Oxoid Ltd, Hampshire, UK). The identification of clinical isolates was carried out on the basis of colony morphology, Gram stain, and API^®^ systems (bioMérieux, Marcy l’Etoile, France). The antimicrobial susceptibility pattern was determined using the Kirby–Bauer disc diffusion method [23]. The antibiotic discs (erythromycin 15 μg, tetracycline 30 μg, netilimicin 30 μg, levofloxacin 5 μg, cefoxitin 30 μg, linezolid 10 μg, rifampicin 30 μg, and gentamicin 10 μg) used for susceptibility tests were supplied by Oxoid Ltd, Hampshire, UK.

*S. aureus* strains were stored at −80 °C before being thawed at room temperature and plated on tryptic soy agar (TSA; Oxoid Ltd, Hampshire, UK). Bacteria were then grown in tryptic soy broth (TSB; Oxoid Ltd, Hampshire, UK) for 24 h at 37 °C under shaking conditions at 125 rpm (Innova 4300, New Brunswick Scientific, Edison, NJ, USA). The overnight broth cultures were resuspended in Mueller–Hinton broth (MHB, Oxoid Ltd, Hampshire, UK) to an optical density at 550 nm (OD_550_) of 1.0 corresponding to 1.0 × 10^8^ colony-forming units (CFU)/mL. The broth cultures were then diluted 1:100 in MHB and used for the evaluation of the minimum inhibitory concentration (MIC) described below, where each well in the 96-well plate contained the bacteria at a final concentration of 1.0 × 10^5^ CFU/mL according to the NCCLS (National Committee for Clinical Laboratory Standards) guidelines [24].

##### 3.2.1.2. Determination of Minimum Inhibitory Concentration (MIC) and Minimum Bactericidal Concentration (MBC)

The MIC and MBC were determined in MHB using the broth microdilution method in 96-well polystyrene microtitre plates (Eppendorf, Hamburg, Germany) according to the NCCLS guidelines. The results obtained were confirmed using the alamarBlue^®^ (AB) (Thermo Fisher Scientific, Waltham, MA, USA) planktonic susceptibility assay [25]. Hemp EO was prepared as 10% (*v*/*v*) solutions in ethanol and used in the range of 0.5–16 mg/mL; naringenin was solubilized in dimethyl sulfoxide (DMSO) and used in the range of 64–1024 μg/mL, while the bacterial concentration was 1.0 × 10^5^ CFU/mL. The plates were incubated at 37 °C for 24 h.

The MIC was defined as the lowest concentration without visible growth. The MBC was defined as the lowest concentration that induced a reduction in CFU corresponding to 99.9% compared to the initial inoculum, and was determined by spreading on MHA with 100 μL of sample taken by the wells corresponding to the MIC. Controls consisting of (i) *S. aureus* broth cultures in MHB without the addition of the sample, (ii) MHB with the test sample at different concentrations, (iii) MHB plus 10% ethanol, and (iv) just MHB were also inserted in the experiments. Three independent experiments were performed in triplicate.

##### 3.2.1.3. AB Planktonic Susceptibility Assay

The experiments were carried out as previously described in Section 3.2.1.2. After the incubation of the plates at 37 °C for 24 h, 10× alamarBlue^®^ (AB) cell viability reagent was added to the treated and untreated broth cultures to assess the viability of bacterial cells. The plates were incubated at 37 °C for 1–4 h. The absorbance was then read at 570 nm (A_570_) and 600 nm (A_600_) on a plate reader (SpectraMax 190, Molecular Devices, San Jose, CA, USA). The percentage reduction of AB in the treated and non-treated samples was calculated using the formula indicated by the manufacturer. Controls consisting of (i) MHB with the test sample at different concentrations, (ii) MHB plus 10% ethanol, and (iii) just MHB were also inserted in the experiments. The AB MIC was defined as the lowest sample concentration resulting in ≤ 50% reduction of AB and a purplish/blue well 1 h after the addition of the AB as reported by Pettit et al. (2005) [25]. Three independent experiments were performed in triplicate.

##### 3.2.1.4. Determination of Minimum Biofilm Eradication Concentration

The antibiofilm effect of each sample was determined by the evaluation of minimum biofilm eradication concentration (MBEC) [26]. The MBEC was defined as the lowest concentration which completely eradicated bacterial biofilm developed in 96-well flat-bottomed polystyrene microtiter plates. Briefly, *S. aureus* was grown in TSB overnight at 37 °C by shaking at 125 rpm. The overnight broth cultures were diluted to 1.0 × 10^5^ CFU/mL in TSB supplemented with 1% (*w*/*v*) glucose. Two hundred microliters of the diluted broth cultures were inoculated into 96-well flat-bottomed polystyrene microtiter plates (Eppendorf, Hamburg, Germany) and incubated at 37 °C for 24 h under static conditions. At the end of incubation, the biofilms were rinsed in phosphate-buffered saline (PBS) and the test sample was added to the pre-formed biofilms at concentrations corresponding to 2 × MIC, 3 × MIC, and 4 × MIC of each test sample. Controls consisting of (i) *S. aureus* biofilms without the addition of the sample, (ii) TSB plus 1% (*w*/*v*) glucose with the sample, (iii) TSB plus 1% (*w*/*v*) glucose plus 10% ethanol, and (iv) just TSB plus 1% (*w*/*v*) glucose were inserted in the experiments. The plates were then incubated at 37 °C for 24 h under static conditions. The inhibitory effect was measured using AB assay, crystal violet (CV) staining, and CFU counts. Three independent experiments were performed in quadruplicate.

##### 3.2.1.5. AB Biofilm Eradication Assay

After the treatment with the test sample, the biofilms were rinsed with PBS, and AB was added following the manufacturer’s instructions. The plates were incubated for 1–4 h at 37 °C. The absorbance was read and the percentage reduction of AB in the treated and non-treated samples was calculated using the formula indicated by the manufacturer. The AB MBEC was defined as the lowest concentration of the test sample resulting in ≤ 50% reduction of AB and a purplish/blue well 1 h after the addition of the AB. Three independent experiments were performed in quadruplicate.

##### 3.2.1.6. Biofilm Eradication Evaluation by Crystal Violet Assay and Live/Dead Cell Viability Staining

The treated and non-treated biofilms were washed with PBS and then dried. Biofilms were stained with 200 μL of 0.5% Gram’s crystal violet and then rinsed with PBS and dried. The biofilm biomass was quantified by destaining the biofilms for 10 min with 33% acetic acid (by volume) and by measuring the absorbance at 590 nm [27]. With regards to the live/dead assay, *S. aureus* biofilms were grown and treated as previously described. The biofilms, treated with 24 mg/mL hemp EO or 2048 μg/mL of naringenin, and the corresponding non-treated controls were rinsed with PBS and stained with a BacLight kit (Thermo Fisher Scientific, Waltham, MA, USA). The analysis was performed using a Leica DMR Fluorescent Microscope (Leica microsystem, Wetzlar, Germany). Three experiments were performed in duplicate.

##### 3.2.1.7. Cell Viability Evaluation through Colony-Forming Unit Count

Colony-forming unit (CFU) enumeration was performed to evaluate bacterial cell viability in the biofilm and planktonic phases. One hundred microliters of sample solutions taken from the MIC and MBEC wells, as well as from the two wells before and after the MIC and MBEC wells, were used for CFU counts. Serial dilutions of the stock were performed in PBS (pH 7.2) and plated on MHA at 37 °C for 18–24 h. The same evaluation was performed in the case of the AB assay because of its non-toxic nature.

#### 3.2.2. Helicobacter pylori

##### 3.2.2.1. Bacterial Strains and Culture Conditions

Fourteen clinical strains of *H. pylori*, with different antibiotic susceptibility patterns, isolated from patients with duodenal ulcer or gastritis, were used for this study. In particular, three strains highly resistant to metronidazole (MNZ; MIC > 8 µg/mL), four resistant to clarithromycin (CLR; MIC > 0.5 µg/mL), four resistant to both MNZ and CLR, and three susceptible to CLR, MNZ, and amoxicillin (AMX) (MIC ≤ 0.125 µg/mL), on the basis of the European Committee on Antimicrobial Susceptibility Testing (EUCAST) breakpoints were classified. A reference strain of *H. pylori* (ATCC 43629) was used as the control. The strains were maintained at −80 °C in Wilkins–Chalgren broth (Difco, BD, San Jose, CA, USA) with 10% (*v*/*v*) horse serum (Seromed, Biocrhom, Germany) and 20% (*v*/*v*) glycerol (Merck, Darmstadt, Germany) until required for experiments. Before being used, the strains were subcultured twice on Columbia agar base (Difco, BD, San Jose, CA, USA) supplemented with 10% horse serum and 0.25% bacto yeast extract (Difco, BD, San Jose, CA, USA). Plates were incubated for 72 h at 37 °C in an atmosphere of 10% CO_2_ in a gas incubator.

##### 3.2.2.2. Minimum Inhibitory Concentration and Minimum Bactericidal Concentration Determination

MICs for Hemp EO and naringenin were determined using a modified broth dilution method as previously described [28]. Briefly, two-fold serial dilutions of the test samples were prepared in a 96-well microtiter plate containing 100 µL of MegaCell™ Roswell Park Memorial Institute (RPMI-1640) medium (Sigma-Aldrich, ST Louis, MO, USA) with 3% fetal calf serum (FCS). An inoculum equivalent to one McFarland standard was prepared in Wilkins–Chalgren broth and diluted in MegaCell™ RPMI-1640 medium with 3% FCS. Each well was inoculated with *H. pylori* at a final concentration of approximately 5 × 10^5^ CFU/well. The plates were incubated at 37 °C under microaerophilic conditions (10% CO_2_ in a gas incubator) and examined after 72 h of incubation. For the MBC, aliquots (10 µL) of suspensions without visible growth were spotted on Columbia agar plates and incubated at 37 °C for 3–5 days under microaerophilic conditions.

#### 3.2.3. *Candida* and *Malassezia* Strain Growth Inhibition

Hemp EO was tested against four clinical isolates of *Candida* spp. (*Candida albicans* CO23, *C. glabrata* DSY 562, *C. krusei* 45709, and C. *tropicalis* 47829) and four clinical isolates of *Malassezia* spp. including *M. furfur* 188 and 180, *M. globosa* 130, and *M. sympodialis* 855. *Candida* strains were cultured in Sabouraud dextrose agar (SDA) at 30–37 °C with aeration for 24 h. *Malassezia* isolates were sub-cultured at 32 ± 2 °C for 72 h on liquid Leeming–Notman medium modified (1% peptone, 0.5% glucose, 0.01% yeast extract, 0.4% ox-bile, 0.2% glycerol, 1% whole cow’s milk, and 0.05% Tween 20). MIC was determined using the microbroth dilution method according to the NCCLS [29] for *Candida* strains and modified to support growth of lipid-dependent yeasts [30] for *Malassezia* strains. Solutions of hemp EO (100 mg/mL) were prepared in RPMI-1640 supplemented with Tween 80 (final concentration of 0.001% *v*/*v*). Briefly, to determine the MIC of EO for *Candida* strains, RPMI-1640 supplemented with 3-(*N*-morpholino)propanesulfonic acid (MOPS) at pH 7 was used. For *Malassezia* spp., the RPMI-1640 medium was supplemented with glucose (1.8%), peptone (1%), ox-bile (0.5%), malt extract (0.5%), Tween 20 (0.5%), Tween 80 (0.05%), and glycerol (1%). The dilution, ranging from 12 to 12480 µg/mL of EO, was prepared in 96-well plates. The inoculum size was about 2.5 × 10^3^ cells/mL. The plates were incubated at 30 °C for 48 h or at 32 °C for 72 h for *Candida* spp. and *Malassezia* spp., respectively.

### 3.3. Cytotoxicity

#### 3.3.1. Cell Lines

The human estrogen-dependent (MCF-7) and triple-negative breast cancer (MDA-MB-468) cell lines were kindly provided by Prof. Eufemi (Dept. of Biochemical Science Rossi Fanelli, Sapienza University of Rome, Italy), while colorectal adenocarcinoma (Caco-2) cells were obtained from ATCC (American Type Culture Collection; Manassas, VA, USA). Furthermore, the extrahepatic cholangiocarcinoma cell line (Mz-ChA-1) from human gallbladder along with nonmalignant, immortalized cholangiocytes (H69) were kindly gifted by Prof. G. Alpini (Texas A&M University, Temple, TX, USA). MCF7, MDA-MB-468, and Caco-2 cells were grown at 37 °C in 5% CO_2_ in Dulbecco’s modified Eagle’s medium, supplemented with sodium pyruvate (1%), fetal bovine serum (10% *v*/*v*), glutamine (2 mM), streptomycin (100 µg/mL), and penicillin (100 U/mL). Mz-ChA-1 cells were maintained at 37 °C in a 5% CO_2_ incubator in Connaught Medical Research Laboratories 1066 medium supplemented with 10% fetal bovine serum (FBS), 1% penicillin, gentamycin, and streptomycin, and 2 mM glutamine. At last, the nonmalignant cholangiocytes H69 were cultured in Dulbecco’s modified Eagle’s medium/Nutrient Mixture F-12 Ham (3:1), supplemented with 1% penicillin and streptomycin, plus the following: 1.8 × 10^−4^ M adenine, 5 μg/mL insulin, 5 μg/mL transferrin, 2 × 10^−9^ M triiodothyronine, 1.1 × 10^−6^ M hydrocortisone, 5.5 × 10^−6^ M epinephrine, 1.64 × 10^−6^ M epidermal growth factor, and 10% FBS [31]. All experiments were performed when cells reached the logarithmic growth phase.

#### 3.3.2. Treatment Protocol

The cultured cells were seeded into 96-well microplates (20,000 cells/well), allowed to grow for 24 h, then treated with the hemp EO for a further 24 h. To perform the assay, progressive dilutions of the hemp EO in ethanol (EtOH; 100% *v*/*v*) were prepared, then assessed at 1% *v*/*v* in the cell medium.

#### 3.3.3. Cytotoxicity Assay

After 24-h incubation, the cytotoxicity of the treatment was measured using the 3-[4,5-dimethylthiazol-2-yl]-2,5-diphenyl tetrazolium bromide (MTT) assay according to previously published methods [32]. The assay was carried out three times and, in each experiment, each concentration was tested almost in triplicate. Cell viability was determined as follows: ((OD treated cells − OD medium control)/(OD untreated cells − OD medium control)) × 100.

### 3.4. Enzyme Inhibitory Activity

The enzyme inhibitory activities of the extracts were calculated as equivalents of the corresponding standard drug per gram of the sample (i.e., galantamine for acetylcholinesterase (AChE) and butyrylcholinesterase (BChE), kojic acid for tyrosinase, orlistat for lipase, and acarbose for α-amylase and α-glucosidase inhibition assays).

#### 3.4.1. Cholinesterase Inhibition

Test solution (50 µL) was mixed with 5,5′-dithiobis(2-nitrobenzoic acid) (DTNB; 125 µL) and enzyme solution (AChE or BChE) solution (25 µL) in Tris-HCl buffer (pH 8.0) in a 96-well microplate and incubated for 15 min at room temperature. The reaction was then initiated with the addition of the corresponding substrates (acetylthiocholine iodide (ATCI) or butyrylthiocholine chloride (BTCl), 25 µL). Similarly, a blank was prepared without the enzyme (AChE or BChE) solution. The absorbances of sample and blank were noted at 405 nm after 10-min incubation at 25 °C. Results are expressed as milligrams of galantamine equivalent per gram of extract (GALAE/g extract) [22].

#### 3.4.2. α-Amylase Inhibition

An aliquot of the test solution (25 µL) was mixed with α-amylase solution (50 µL) in phosphate buffer (pH 6.9 with 6 mM sodium chloride) in a 96-well microplate and incubated for 10 min at 37 °C. The reaction was then initiated with the addition of starch solution (50 µL, 0.05%). Similarly, a blank was prepared without the enzyme. The reaction mixture was incubated 10 min at 37 °C, stopped with the addition of HCl (25 µL, 1 M), and then the iodine/potassium iodide solution was added (100 µL). The absorbances of sample and blank were calculated at 630 nm. The absorbance of the blank was subtracted from that of the sample. Results are expressed as millimoles of acarbose equivalent per gram of extract (ACAE/g extract) [33].

#### 3.4.3. α-Glucosidase Inhibition

Test solution (50 µL) was mixed with glutathione (50 µL), α-glucosidase solution (50 µL) in phosphate buffer (pH 6.8), and *p*-nitrophenyl-β-d-glucuronide (PNPG; 50 µL) in a 96-well microplate and incubated for 15 min at 37 °C. Similarly, a blank was prepared without the enzyme solution. The reaction was stopped with sodium carbonate (50 µL, 0.2 M) and the absorbances of sample and blank were noted at 400 nm. Results are expressed as millimoles of acarbose equivalent per gram of extract (ACAE/g extract) [33].

#### 3.4.4. Tyrosinase Inhibition

Test solution (25 µL) was mixed with tyrosinase solution (40 µL) and phosphate buffer (100 µL, pH 6.8) in a 96-well microplate and incubated for 15 min at 25 °C. The reaction was then initiated with l-3,4-dihydroxyphenylalanine (l-DOPA; 40 µL). Similarly, a blank was prepared (without the enzyme solution). The absorbances of sample and blank were recorded at 492 nm after 10-min incubation at the room temperature. Results are expressed as milligrams of kojic acid equivalent per gram of extract (KAE/g extract) [34].

#### 3.4.5. Lipase Inhibition

Porcine pancreatic lipase (type-II) activity was performed using *p*-nitrophenyl butyrate (*p*-NPB) as a substrate [35]. The enzyme solution (1 mg/mL) was prepared in 50 mM Tris-HCl (pH 8.0). An aliquot of 25 µL was mixed with a lipase solution (50 µL) in a 96-well microplate and incubated for 20 min at 25 °C. The reaction was initiated with the addition of *p*-NPB (5 mM, 50 µL). Similarly, a blank sample (prepared in the same manner but without the extract) was prepared and analyzed according to this procedure. Results are expressed as milligrams of orlistat equivalent per gram of extract (OE/g extract).

### 3.5. *G. mellonella* Injection Procedure

Ten larvae (250–320 mg each) of *Galleria mellonella* were selected at random for each step in the procedure. Different concentrations from 0.78 to 6.25 mg/mL hemp EO in PBS buffer added with 0.01% Tween 20 were injected into the hemocoel through the last left proleg (Hamilton syringe 701N; volume, 10 μL; needle size, 26 s; cone tip; Sigma-Aldrich, Milan, Italy) [36]. After injection, larvae were incubated in petri dishes at 37 °C in standard aerobic conditions and survival was recorded at 24-h intervals for five days. Larvae were considered dead when they displayed no movement in response to gentle prodding with a pipette tip. Studies included one group that did not receive injection and one group that was injected with PBS plus 0.01% Tween 20 as a control. Each experiment was repeated three times.

### 3.6. Statistical Analysis

The statistical significance of difference between controls and experimental groups for the antimicrobial activity was evaluated using Student’s *t*-test. Results of the anti-proliferative activity were expressed as means ± standard error of the mean (SEM). Statistical analysis was performed using GraphPad Prism™ (Version 5.00) software (GraphPad Software, San Diego, CA, USA). The one-way analysis of variance (one-way ANOVA), followed by Dunnett’s multiple comparison post hoc test, was used to verify the significance level of a response with respect to the vehicle control. A *p*-value < 0.05 was considered as statistically significant.

## 4. Results and Discussion

The hemp crop produced approximatively 10,000–12,000 kg/ha of fresh biomass. The hydro-distillation of flowering aerial parts gave 0.28% essential oil yield (mL/kg fresh weight), expressed as the mean value of separate distillations from 2 kg of fresh weight each. Minimum and maximum ranges of EO were 0.19% and 0.31%, respectively. The highest yield value was obtained in the early-September distillation, while the lowest was from the late-September/early-October collection (full blooming plants) (Figure S1, Supplementary Materials). As expected, the difference in EO yield was not evident in day-by-day analysis, while there was a significant difference (* *p* < 0.05) in the data comparison between the collections on the first and last days (0.29 ± 0.02% and 0.19 ± 0.01% mean yield ± SEM, 1 September and 2 October, respectively). The trend of yield variation was also evident upon analyzing data grouped by decades, confirming the early collection as the preferable practice for optimal yield (0.29 ± 0.004%, 0.25 ± 0.005%, and 0.21 ± 0.003% mean yield ± SEM; 1–11 September, 12–22 September, and 23 September–2 October, respectively (ANOVA, *p* < 0.0001; *** *p* < 0.001). The quantitative production of hemp EO was monitored for one month. Fresh plant material was collected daily, manually selecting plants only in full bloom, and three or four separated distillations were immediately performed. Experimental data confirm that plant harvest is preferable at the early flowering stage due to the evident reduction in EO content in proximity to fruiting. From the weekly data analysis, the reduction (−30%) of EO production was evident in late flowering stages, while all yield data within the same week were more homogenous. At the end of the production process, all collected hemp EO samples were pooled to provide a single batch available for the market and for the successive analyses. The aromatic water, separated from the EO but enriched by fragrant molecules, was further investigated for its chemical composition, antioxidant activity, color characteristics, and enzyme inhibitory activity with the aim of giving value to this important by-product.

### 4.1. Phytochemical Analyses

#### 4.1.1. Color Analysis

**Figure 1:**
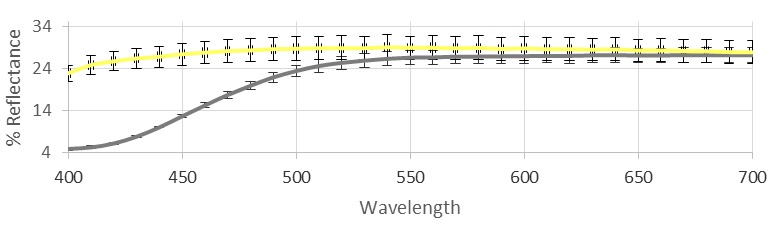
Reflectance curves of hemp essential oil (EO; gray) and its aromatic water (yellow).

The tristimulus colorimetry was not yet reported in the literature to evaluate the color properties of hemp EO and its aromatic water. The EO, obtained from the fresh inflorescences via steam distillation and separated by water, mainly contains a mixture of monoterpene and sesquiterpene hydrocarbons. Our analyses, as subsequently described, account for twelve components belonging to this class, in which (*E*)-caryophyllene and caryophyllene oxide represent the principal impact compounds. As a counterpart in the aromatic water, a polyphenolic fraction mainly represented by catechins and phenolic acids was found. The color of the EO, which could vary between pale yellow and light green was found to show a significant yellow parameter (b*) along with a weak green value (a*), which, together with the quite bright L* value, account for the light yellow-green nuance characterizing our sample. The aromatic water showed a very clear yellow color associated with a relatively high L* value and particularly low a* and b* parameters. If this is related to the catechin content, the more intense yellow-green color of the separated oil could be maybe attributed to other pigments, weakly represented but intensely colored, such as chlorophylls, carotenoids, or others [37]. The colorimetric data, complete with standard deviations, are reported in Table 1, and the reflectance curves are shown in Figure 1.

#### 4.1.2. GC Flame Ionization Detector Analysis of the Tetrahydrocannabinol (THC) Content

The THC content was routinely checked on *C. sativa* var. Futura 75 aerial parts and on the processed final product (hemp EO) and was found to be below the limits of Italian government law (THC < 0.2%).

#### 4.1.3. GC/MS Analysis of the Volatile Components of the EO

The GC/MS analysis of hemp EO exhibited several peaks that were related to different classes of phytochemicals. The most abundant components belonged to the class of sesquiterpenes and sesquiterpenoids (28% (*E*)-caryophyllene, 4% *trans*-α-bergamotene, 13% humulene, 7% α- and β-selinene, and 15% caryophyllene oxide) representing about 67% of the total composition (Table 2). Monoterpenoids were also present in an appreciable amount (11% total pinene fraction and 11% β-myrcene), together with lower-abundance components ranging from 6% (α-terpinolene) to 2% (d-limonene).

#### 4.1.4. Total Phenolic, Flavonoid, and Phenolic Acid Content

The amounts of total bioactive compounds in the aromatic water were detected by colorimetric methods, and the results are summarized in Table 3 (hemp EO was not soluble in the assay reagents). Total phenolics, flavonoids, and phenolic acids were found to be 28.04 mg GAE/g, 4.04 mg RE/g, and 1.76 mg CE/g extract, respectively. Apparently, the total amount of flavonoids and phenolic acids was approximately 20% of total phenolics. Several researchers reported different levels of total bioactive compounds in *Cannabis sativa* extracts [38,39,40,41,42] and, generally, the extracts were found to be rich sources of these compounds. These differences in the literature may also be linked to geographical and climatic conditions, or genetic structures.

#### 4.1.5. HPLC-PDA Analysis of the Phenolic Fraction

Both hemp EO and its aromatic water, studied for the presence of the most important 22 phenolics, displayed an interesting profile in terms of richness and metabolite-oriented amount (Table 4) [43].

Naringenin (706 µg/mL) and its glycosylated derivative naringin (83 µg/mL) emerged, among the ones studied, as the two most important metabolites which could further characterize this EO, along with the presence of catechin (60 µg/mL) and epicatechin (56 µg/mL) (see Supplementary Materials for the chromatogram of a real sample). Considering the mean concentration of naringenin, hemp EO seems to be relevant as a source of this important flavanone for cosmetic and pharmaceutical purposes. Moreover, naringenin was shown to act as an antioxidant, anti-inflammatory, anti-cancer, anti-diabetic, and anti-obesity agent [44]. Other compounds such as benzoic acid, 2,3-dimethoxybenzoic acid, and syringic acid were also detected in discrete quantities. Conversely, the number and the relative amounts of these phytochemicals were scarcely found in the aromatic water (rich in catechin), despite the unique presence of a small percentage of rutin (0.18 µg/mL). Chlorogenic acid, vanillic acid, 3-hydroxy-4-methoxybenzaldehyde, *p*-coumaric acid, sinapinic acid, *o*-coumaric acid, harpagoside, *t*-cinnamic acid, and carvacrol were absent in this EO and in its aromatic water.

### 4.2. Antioxidant Properties

Antioxidative effects of natural products could be considered as a first insight in detecting their ethnopharmacological relevance and potential. To this end, a certain antioxidant profile could be provided by comparing different chemical assays representative of alternative mechanisms. For this reason, the antioxidant ability of *C. sativa* samples was evaluated using six complementary in vitro tests: free-radical scavenging (DPPH and ABTS), reducing power (CUPRAC and FRAP), phosphomolybdenum, and metal chelating assays. The results are tabulated in Table 5. The aromatic water exhibited stronger free-radical scavenging and ferric reducing potential when compared to the EO. In addition, the EO was not active on ABTS radicals. However, the best activity in phosphomolybdenum and metal chelating assays was obtained by the EO. These findings may be linked to the presence of a large amount of naringenin and catechin, which act as good chelators [45,46]. Also, the phosphomolybdenum assay is known as a total antioxidant test, and different phytochemicals, including vitamin C, tocopherol, or carotenoids, may be active in this assay. CUPRAC and FRAP tests showed good results with these products, which, along with metal chelating activity, could account for the potential of hemp EO in limiting the generation and the accumulation of harmful free radicals via the Fenton reaction. Similarly, some researchers [39,40,47,48] reported that *C. sativa* has great potential as a source of natural antioxidants for developing novel functional applications.

### 4.3. Antimicrobial and Antibiofilm Activity of the Hemp EO versus *Staphylococcus aureus*

*Staphylococcus aureus* is a Gram-positive bacterium which permanently or transiently colonizes up to 60% of the human population and, for this reason, is also considered a component of the human microbiota [49]. *S. aureus*, however, is associated with nosocomial infections including those involving orthopedic implants [50], and may be responsible for many infections such as endocarditis, osteomyelitis, prostatitis, and infections of skin and soft tissue [51]. Its persistence and recalcitrance are due to the capability of *S. aureus* to develop biofilms as a complex structure formed by bacterial cells embedded in an extracellular polymeric substance (EPS), a mixture of macromolecules such as exopolysaccharides, proteins, and extracellular DNA. The EPS composition may differ with bacterial strain, culture conditions, and biofilm age. Biofilm formation guarantees antibiotic tolerance and protection from the host immune system, making microbial biofilms difficult to eradicate [52].

The inhibitory effect of hemp EO against Gram-positive opportunistic/pathogenic microorganisms such as *Clostridium* spp. and *Enterococcus* spp. was demonstrated [5], suggesting its use against nosocomial, spoilage, and food-borne pathogens. In our paper, the antimicrobial and antibiofilm effects of hemp EO were evaluated against five *S. aureus* strains, one reference and four clinical strains, isolated from different areas of the body and also characterized by a different antimicrobial susceptibility pattern (Table 6; Table S1, Supplementary Materials).

**Figure 2:**
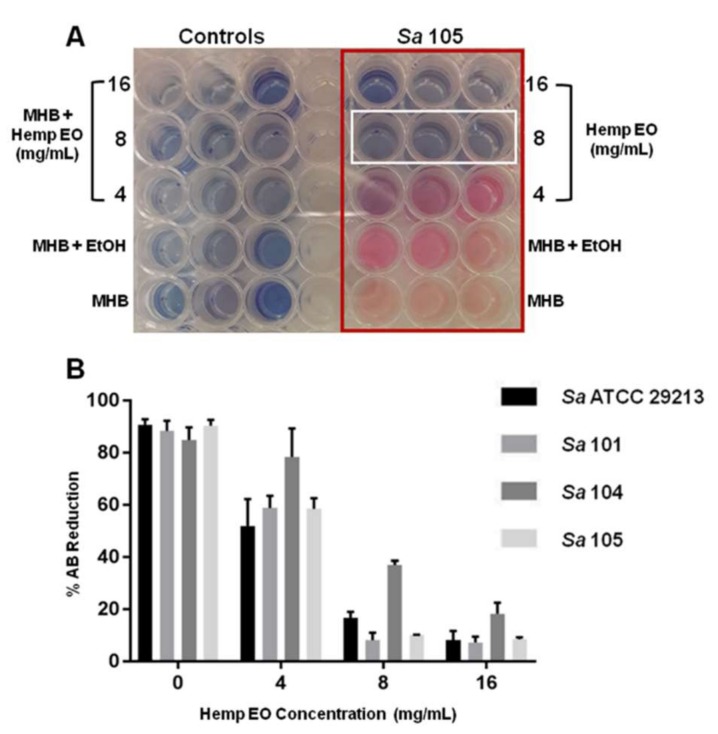
The evaluation of minimum inhibitory concentration (MIC) of the hemp EO versus *Staphylococcus aureus* determined using the alamarBlue^®^ (AB) assay. (**A**) Representative image of colorimetric MIC determination using AB at 24 h of incubation. The white rectangle indicates the MICs at 8 mg/mL *S. aureus* 101. (**B**) The plot shows the percentage reduction of AB in the *S. aureus* broth cultures at different hemp EO concentrations compared to the corresponding untreated samples (0) evaluated as indicated in the experimental section of this manuscript. MHB: Mueller–Hinton broth; EtOH: ethanol. Data are presented as the mean of three replicates of three independent experiments.

The hemp EO showed MIC values corresponding to 8 mg/mL versus all *S. aureus* strains, including *S. aureus* 104, a multi-drug resistant strain isolated from a pharyngeal swab of a male patient (Table 6). With regards to MBC, the hemp EO was shown to be effective at 16 mg/mL versus all *S. aureus* strains (Table 6). The results obtained were confirmed using the AB reduction assay (Figure 2A,B).

**Figure 3:**
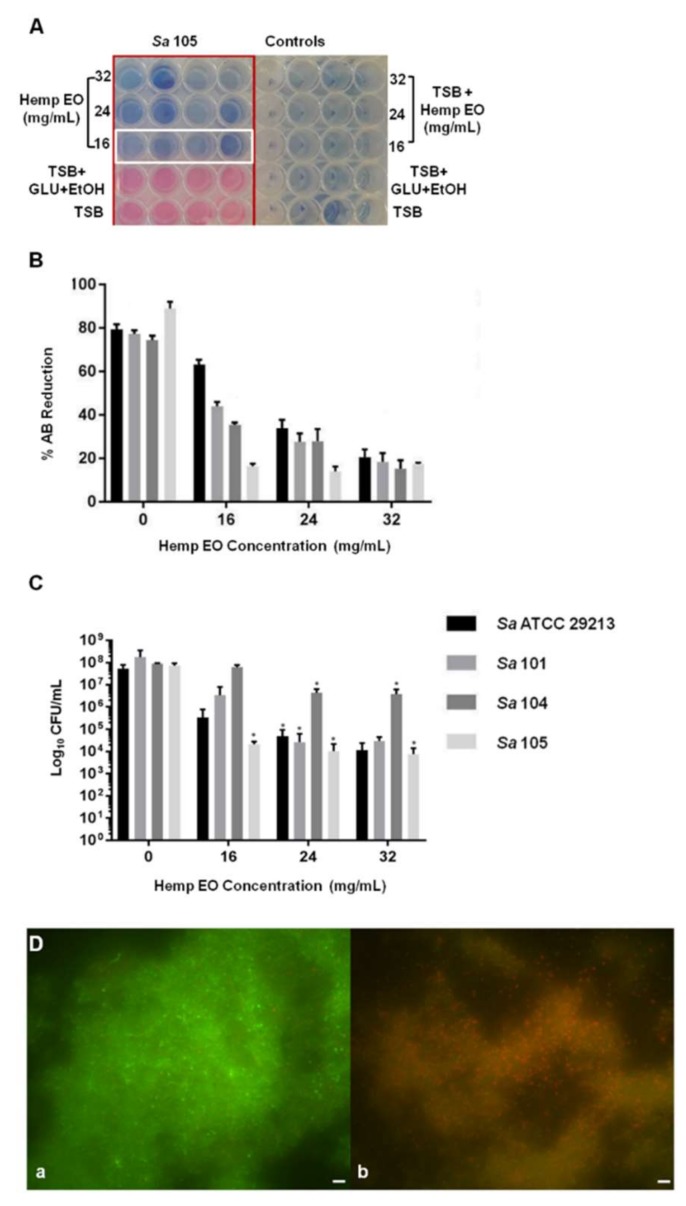
The evaluation of minimum biofilm eradication concentration (MBEC) of the hemp EO versus *Staphylococcus aureus* biofilm determined using the AB assay, live/dead staining, and fluorescent microscopy analysis and colony-forming unit (CFU) counts. (**A**) Representative image of colorimetric MBEC determination using AB. The white rectangles indicate the MBECs at 24 mg/mL *S. aureus* 105. (**B**) The plot shows the percent reduction of AB in the *S. aureus* broth cultures at different hemp EO concentrations compared to the corresponding untreated samples (0) evaluated as indicated in Section 2. (**C**) CFU count of treated and untreated *S. aureus* biofilms. (**D**) Representative image of *S. aureus* (**a**) untreated and (**b**) treated biofilms with 24 mg/mL hemp EO. The biofilms were stained with a live/dead kit and visualized using fluorescent microscopy. The green fluorescence indicates live cells, whereas the red fluorescence indicates dead cells or cells with a compromised cell wall. TSB: tryptic soy broth; GLU: glucose. Data are presented as the mean of three replicates of three independent experiments; * *p* ˂ 0.05 vs. the controls (0).

Furthermore, the hemp EO showed its capability to eradicate a mature biofilm developed by *S. aureus*; specifically, the minimum biofilm eradication concentration (MBEC) was determined using the AB assay, CV staining, CFU counting, and live/dead staining, followed by fluorescent microscopy analysis. The Hemp EO displayed an MBEC value of 24 mg/mL toward all strains of *S. aureus* except for *S. aureus* 105, which showed an MBEC value corresponding to 16 mg/mL (Table 6 and Figure 3A,B). The effect of the hemp EO on the eradication of *S. aureus* pre-formed biofilms was confirmed by the statistically significant reduction in CFU count, as shown in Figure 3C. The live/dead staining and fluorescent microscopy evaluation showed a well-structured biofilm constituting a great amount of live cells, as indicated by the green fluorescence (Figure 3D(a)). On the contrary, *S. aureus* mature biofilms treated with 24 mg/mL hemp EO did not show a reduction in biofilm biomass, as confirmed by the CV staining (data not shown); however, the biofilms treated with hemp EO were characterized by a multitude of dead cells as indicated by the red fluorescence (Figure 3D(b)). As mentioned above, the CV staining did not display any differences between the biomasses of treated and untreated biofilms (data not shown).

Due to the high amount of naringenin in this EO, we aimed to evaluate the impact of this secondary metabolite on the antimicrobial activity. We also studied the MIC, MBC, and MBEC of naringenin against *S. aureus* 105. The data indicated an MIC of 512 μg/mL and an MBEC corresponding to 2048 μg/mL. The results demonstrated the antimicrobial and antibiofilm effects of naringenin contained in the hemp EO versus *S. aureus*. These results also confirmed the data of Yue et al. [53] who reported the effect of naringenin on the formation of the biofilm of *Streptococcus mutans*. The authors demonstrated that the MIC of naringenin was between 100 and 200 μg/mL, and that 200 μg/mL naringenin was capable of inhibiting the biofilm formation after 4 and 24 h of incubation. Conversely, in the present study, we focalized the attention on the capability of naringenin to eradicate a mature biofilm developed by *S. aureus* after 24 h of incubation to evaluate an application of hemp EO in the treatment of pre-formed biofilms.

Moreover, the antimicrobial effect of naringenin was also previously investigated on several bacterial species. In particular, the mechanism of action of naringenin was studied on *Escherichia coli* ATCC 25922 and *S. aureus* ATCC 6538. The authors demonstrated that the presence of naringenin induced modifications of cell-membrane fluidity, fatty-acid composition, and fatty-acid biosynthesis-associated genes. Furthermore, the growth of *S. aureus* was significantly inhibited by low concentrations of naringenin [54,55].

### 4.4. Anti-Helicobacter Pylori Activity

The antimicrobial potency of hemp EO and its main component naringenin was also explored against 14 *H. pylori* clinical isolates and the reference strain ATCC 43629, using the microdilution method at pH 7.5 [56]. As shown in Table 7, the activity of the two test samples was not related to the different antibiotic susceptibility of the strains; the EO showed an MIC_50_ of 32 µg/mL and an MIC_90_ of 64 µg/mL, whereas naringenin showed an MIC_50_ of 16 µg/mL and an MIC_90_ of 32 µg/mL. By considering the strains resistant to the traditional antibiotics (MNZ and CLR), hemp EO was 2–4-fold more active than MNZ, and 4–16-fold more active than CLR. The potency of naringenin was 2–8- and 4–16-fold higher than MNZ and CLR, respectively. MBC values were 64 µg/mL for hemp EO and 32 µg/mL for naringenin. The MIC and MBC concentrations of all strains were the same for both compounds, except for the strains E34, 68, and F40/442, which had MBC values corresponding to two times the MIC. Because a drug is defined “bactericidal” when the MBC concentration does not exceed four-fold MIC value [57], we can conclude that hemp EO and naringenin have very good bactericidal effects (1 < MBC/MIC < 2).

Nariman and coworkers [58] reported a study about the anti-*Helicobacter pylori* effect of *C. sativa* and other 19 plants extracts, collected from Iran areas. Of the 10 strains tested, six were defined susceptible by disc diffusion method. The MIC values of the 10 most effective extracts (*C. sativa* not included) against *Helicobacter pylori* ranged from 31.25 to 500 µg/mL. Following these studies and the threshold values reported by Kuete (2010) [59], our results demonstrate that hemp EO and naringenin have very good activity against *H. pylori.* Furthermore, it was reported that cannabidiol prevents intestinal inflammation damage induced by an alteration of cytokine levels [60]. Because the pathogenic effect of *H. pylori* infection is supposed to also be mediated by cytokine production of inflammatory cells present in the infected gastric mucosa [61], the antimicrobial activity of *C. sativa* extracts demonstrated in this study, associated with their anti-inflammatory effect, could represent a very interesting approach against *Helicobacter pylori* infection.

### 4.5. Antifungal Activity of Hemp EO

The antifungal activity of hemp EO against *Candida* spp. and *Malassezia* spp. was calculated as MIC value >12,460 µg/mL with no differences observed among *Candida* spp. and *Malassezia* spp. Hemp EO was not active against yeasts following the defined threshold activity values for plant extracts proposed as follows: MIC below 100 μg/mL (significant activity), 100 ≤ MIC ≤ 625 μg/mL (moderate activity), and MIC > 625 μg/mL (low activity) [59]. These results reinforced the selective antibacterial activity of hemp EO.

### 4.6. Cytotoxicity

**Figure 4:**
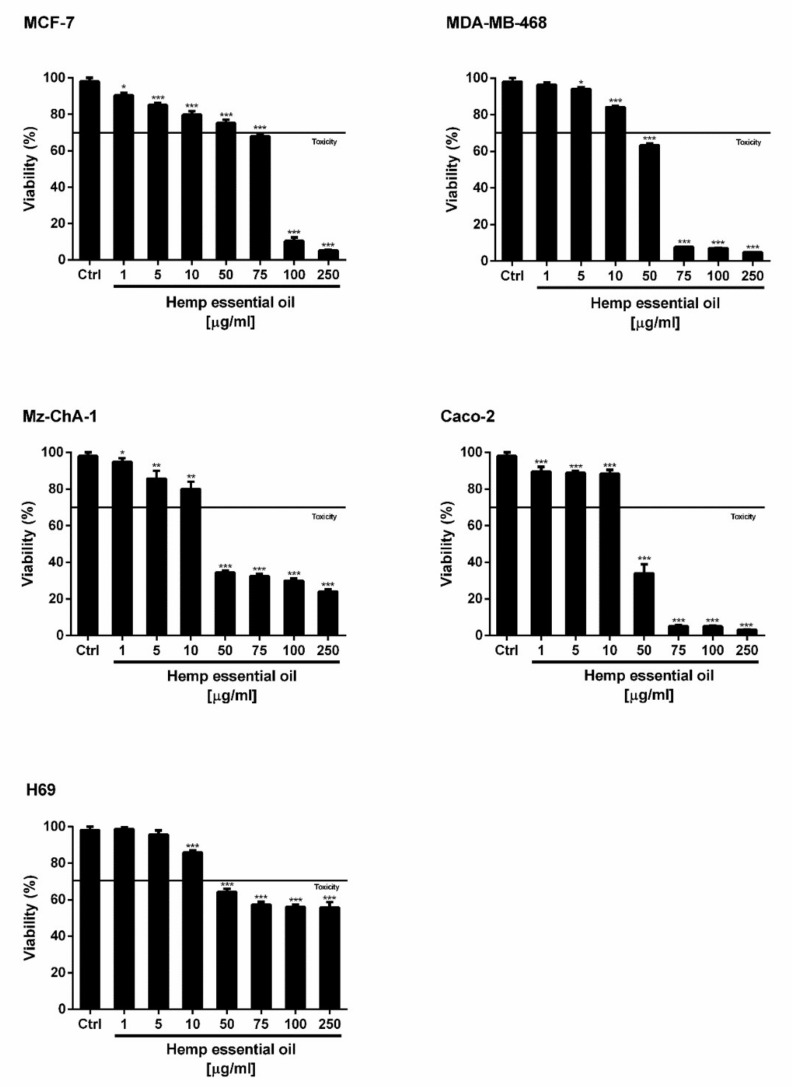
Cytotoxic effect of hemp EO in different human cell lines after 24 h of exposure. MCF-7, estrogen-dependent breast cancer cells; MDA-MB-468, triple-negative breast cancer cells; Caco-2, colorectal adenocarcinoma cells; Mz-ChA-1, cholangiocarcinoma cells; H69, nonmalignant immortalized cholangiocytes. Data are the mean ± SEM from almost two independent experiments with three technical replicates (*n* = 6); * *p* < 0.05, ** *p* < 0.01, and *** *p* < 0.001 vs. control determined by ANOVA followed by Dunnett’s multiple comparison post hoc test.

To the best of our knowledge, the cytotoxicity of hemp EO in cancer cells is yet to be investigated. Under our experimental conditions, the hemp EO induced a statistically significant and concentration-dependent reduction of cancer cell viability, reaching almost a 75% inhibition of cell proliferation at the highest concentration of 250 µg/mL (Figure 4). A different anti-proliferative potency was found in the tested cell models, with Caco-2 and Mz-ChA-1 cells as the most sensitive biological systems. In fact, after 24 h of treatment with 50 µg/mL hemp EO, the cell viability was inhibited by about 66% in both cell lines (Figure 4). Accordingly, similar half maximal inhibitory concentration (IC_50_) values were calculated for Caco-2 and Mz-ChA-1 cells (Table 8).

Next, hemp EO significantly inhibited the growth of both breast cancer cell lines characterized by different biological features and heterogeneous susceptibility to anticancer agents, with more potency against MDA-MB-468 cells. In particular, the treatment with 50 and 75 µg/mL produced an inhibition of cell viability of 25% and 32% in MCF7 cells, and of 37% and 92% in MDA-MB-468 cells; 90% proliferation inhibition was reached only at 100 µg/mL in MCF7 cells. As expected, the resulting IC_50_ value of the Hemp EO in MCF7 cells was about 1.6-fold higher than that obtained for MDA-MB-468 cells, and almost threefold higher than that calculated for Caco-2 and Mz-ChA-1 cells. In contrast, slight cytotoxicity was produced in the nonmalignant cholangiocytes (H69), reaching a maximum of 44% inhibition of cell proliferation at the highest concentration of 250 µg/mL; as a consequence, the IC_50_ was not evaluable. On the basis of these results, the cytotoxicity of hemp EO appeared to be selective for cancer cells, particularly cholangiocarcinoma and coloncarcinoma, with a higher tolerability in normal cells. The cytotoxicity was similar to that of the positive control doxorubicin in Caco-2 and Mz-ChA-1 cells, while about 10-fold lower in breast cancer MCF7 and MDA-MB-468 cells (Table 8). Of note, this behavior suggests that hemp EO can affect specific cancer targets, which deserves to be deeply investigated.

Upon phytochemical analysis, several terpenoids, including monoterpenes and sesquiterpenes, along with flavonoids and tannins, were identified in this phytocomplex. Interestingly, α-pinene and β-caryophyllene exhibited high anti-proliferative activity on human erythroleukemic K562 cells [62]; moreover, β-caryophyllene and linalool exhibited interesting anti-proliferative activity on human amelanotic melanoma C32 cells and on renal cell adenocarcinoma cells [63]. β-Caryophyllene was also reported to possess chemopreventive properties and to interfere with liver cancer cell proliferation by inhibiting the activation of signal transducer and activator of transcription 3 (STAT3) signaling [64], whereas β-caryophyllene oxide was found to possess anti-proliferative activity, although at high concentrations, and chemosensitizing properties, thus restoring the sensitivity of resistant cancer cells to chemotherapy [65]. β-Myrcene was reported to possess anti-invasive properties in metastatic MDA-MB-231 human breast cancer cells through the inhibition of nuclear factor kappa B (NF-κB) activity [66]. d-Limonene is widely known to possess chemopreventive and antitumor properties; in a previous study, it induced tumor regression and cancer cell apoptosis, and inhibited inflammation and oxidative stress [67].

Among phenolics, naringin was reported to possess antitumor properties and to inhibit the growth of gastric cancer cells through the induction of autophagy [68]. The anti-proliferative and pro-apoptotic activity of naringenin is also well documented [69,70]. This evidence suggests that several phytoconstituents can represent the possible bioactive agents of hemp EO, although the role of unique interactions of all the compounds in the phytocomplex cannot be excluded.

### 4.7. Enzyme Inhibition

Enzyme inhibitory effects of the tested *C. sativa* samples were investigated toward a panel of important enzymes such as acetylcholinesterase (AChE), butyrylcholinesterase (BChE), α-amylase, α-glucosidase, tyrosinase, and lipase. The results are presented in Table 9. Generally, from the enzymatic standpoint, the highest values were observed in the EO, except for the lack of inhibitory effect on AChE and α-amylase. These variations observed can be explained by the different phytochemical compositions of the *C. sativa* samples. For example, the EO is richer in terms of volatile components, such as (*E*)-caryophyllene, caryophyllene oxide, and humulene, which are known as anti-tyrosinase agents [71]. Similarly, naringenin, the major compound in the EO, is very active on α-glucosidase and pancreatic lipase [72,73]. In particular, lipase inhibition was the most promising effect after treatment with hemp EO.

In addition, the complex nature of phytochemicals or their interactions could be responsible for the observed inhibitory activities. Up to now, few studies were carried out on the enzyme inhibitory effect of *C. sativa* [42,74,75]. With this regard, the present study could provide a starting contribution for the scientific audience, and this plant may serve to prepare functional ingredients as a source of natural inhibitors.

### 4.8. In Vivo Toxicity Studies

**Figure 5:**
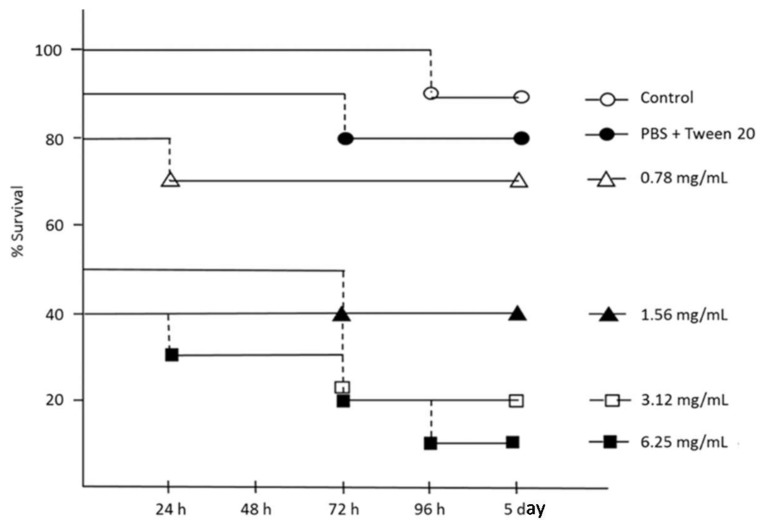
Survival curves of *Galleria mellonella* larvae after hemp EO treatment. Each data point represents the percent survival of *G. mellonella* larvae (*N* = 10/group, repeated on three different occasions), following injection with test samples and incubation at 37 °C. Control larvae were not injected or injected with phosphate-buffered saline (PBS) plus Tween 20 to simulate the trauma associated with the administration. Larvae were monitored for 24 h up to five days.

Larvae of *Galleria mellonella* are widely used as an invertebrate wax moth larva model to evaluate the virulence of microbial pathogens, measure the efficacy/safety of biologically active antimicrobial agents, and produce results comparable to those that can be obtained using mammals [76]. As a consequence, no ethical approval was required for this study because there was no use of mammal or animal models. In this work, the viability of *G. mellonella* larvae, to measure the relative toxicity of different concentrations of hemp EO, was evaluated over the study period of five days. As reported in Figure 5, no evident effects on survival were registered with 0.78 mg/mL hemp EO up to five days after treatment. The lethal dose (LD_50_) of Hemp EO was 1.56 mg/mL in the first 48 h of treatment, probably due to the lipophilic nature of the EO with respect to the larval biological fluids and the invasive administration procedure (Figure 5).

The LD_50_ of 1.56 mg/mL was higher than the effective concentrations reported by us against *H. pylori* (8–64 μg/mL) and cell lines (22.3–250 μg/mL), but in the same range as those found active against *S. aureus* strains (8–24 mg/mL). In this case, the putative topical administration of the hemp EO to combat *S. aureus* wound infections guarantees a discrete safety index with respect to the parental administration in *G. mellonella* larvae.

## 5. Conclusions

The study of traditional crops could explain their ethnobotanical relevance by means of a deep analysis of the quali-quantitative phytochemical profile. We devoted our attention to the EO of the aerial parts of *C. sativa* var. Futura 75 cultivated in Italy, due to an increment in this crop’s cultivation. After the evaluation of tetrahydrocannabinol content, volatile fraction, phenolic and flavonoid pattern, and color parameters, we tested the EO of *C. sativa* aerial parts and its aromatic water against several targets of pharmaceutical interest (*S. aureus*, *H. pylori*, *Candida* and *Malassezia* spp., enzymes, and cancer cell lines), along with the preliminary evaluation of its safety toward a non-cancer cell line and *G. mellonella* larvae in vivo. In addition, due to the high content of naringenin, we further explored the inhibitory effects of this secondary metabolite against *H. pylori* and *S. aureus* to correlate the biological activity with the chemical composition. In particular, the antibacterial and antibiofilm activities of hemp EO suggested it could be a possible candidate for the treatment of infections related to those abovementioned microorganisms. Currently, reinvigorating the antimicrobial drug pipeline is mandatory, as increasingly resistant species continue to emerge or organize themselves in accessible communities. Secondary metabolites display drug-like and metabolite-like properties, acting synergistically with validated drugs, and possessing new mechanisms of action; nevertheless, the evaluation of their safety/tolerability is strongly requested.

## Figures and Tables

**Table 1:** Colorimetric data of hemp essential oil (EO) and its aromatic water.

CIELAB Parameters *	Hemp Essential Oil Mean Value ± SD	Aromatic Water Mean Value ± SD
L*	57.26 ± 1.66	60.46 ± 2.54
a*	−5.68 ± 0.33	−1.22 ± 0.15
b*	24.36 ± 1.63	2.23 ± 0.24
C*_ab_	25.01 ± 1.66	2.54 ± 0.28
*h_ab_*	103.13 ± 0.11	118.66 ± 0.72

* CIELAB parameters (L*, a*, b*, C*_ab_ and *h*_ab_) are defined as follows: L* defines the lightness and varies between 0 (absolute black) and 100 (absolute white), a* measures the greenness (−a*) or the redness (+a*) and b* measures the blueness (−b*) and the yellowness (+b*). C*_ab_ (chroma, saturation) expresses a measure of color intensity and *h*_ab_ (hue, color angle) is the attribute of appearance by which a color is identified according to its resemblance to red, yellow, green, or blue, or a combination of two of these attributes in sequence.

**Table 2:** Phytochemicals identified by GC/MS analysis of hemp EO.

Compound	Area %	KI ^b^	KI_lit_^c^
α-Pinene ^a^	8	936	936
β-Pinene	3	975	978
β-Myrcene ^a^	11	984	987
d-Limonene ^a^	2	1030	1027
β-Ocimene	3	1051	1050
α-Terpinolene	6	1089	1084
(*E*)-Caryophyllene ^a^	28	1427	1427
*trans*-α-Bergamotene	4	1439	1437
Humulene	13	1459	1459
β-Selinene	4	1492	1486
α-Selinene	3	1500	1497
Caryophyllene oxide ^a^	15	1592	1589

^a^ Standards available from commercial sources were injected to further confirm the assignment. ^b^ The Kovats index (KI) values were experimentally measured using *n*-alkanes mixtures (C8–C24) on the HP-5MS column. ^c^ Literature values.

**Table 3:** Total bioactive compounds in *Cannabis sativa* extract samples.

Test Sample	Total Phenolic Content (mg GAE/g Extract) *	Total Flavonoid Content (mg RE/g Extract) *	Total Phenolic Acid Content (mg CE/g Extract) *
Hemp EO	nt	nt	nt
Aromatic water	28.04 ± 0.23	4.04 ± 0.03	1.76 ± 0.14

* Values expressed are means ± SD of three parallel measurements. GAE: gallic acid equivalent; RE: rutin equivalent; CE: caffeic acid equivalent; nt: not tested due to solubility issues.

**Table 4:** Quantitative (µg/mL) * multicomponent phenolic pattern in test samples.

Compound	Hemp EO	Aromatic Water
Gallic acid	0.23 ± 0.03	0.62 ± 0.08
Catechin	60 ± 4	7.5 ± 0.2
*p*-OH Benzoic acid	0.35 ± 0.02	
Epicatechin	56 ± 5	
Syringic acid	7.8 ± 1.3	
3-OH Benzoic acid	4.6 ± 0.4	
Rutin		0.18 ± 0.03
*t*-Ferulic acid	0.37 ± 0.04	
Naringin	83 ± 15	0.63 ± 0.09
2,3-DiMeO benzoic acid	10.4 ± 0.3	
Benzoic acid	31.9 ± 0.9	
Quercetin	1.7 ± 0.1	
Naringenin	706 ± 62	0.16 ± 0.02
Total	962.35	9.09

* Data reported are means ± SD from three independent determinations.

**Table 5:** Antioxidant properties * of the *C. sativa* samples.

Test Sample	Phosphomolybdenum (mmol TE/g Extract or Oil)	DPPH (mg TE/g Sample)	ABTS (mg TE/g Sample)	CUPRAC (mg TE/g Sample)	FRAP (mg TE/g Sample)	Metal Chelating Activity (mg EDTAE/g Sample)
Hemp EO	35.12 ± 1.63	5.56 ± 0.42	na	141.15 ± 2.74	57.02 ± 0.69	19.27 ± 1.23
Aromatic water	1.42 ± 0.05	39.71 ± 1.54	103.38 ± 0.07	109.15 ± 1.78	82.93 ± 1.70	3.79 ± 0.25

* Values expressed are means ± SD of three parallel measurements. GAE: gallic acid equivalent; RE: rutin equivalent; CE: caffeic acid equivalent; TE: trolox equivalent; EDTAE: ethylenediaminetetraacetic acid (EDTA) equivalent; na: not active.

**Table 6:** The evaluation of the minimum inhibitory concentration (MIC), minimum bactericidal concentration (MBC), and minimum biofilm eradication concentration (MBEC) of the hemp EO against *Staphylococcus aureus.* Data are presented as the mean of three replicates of three independent experiments. ATCC—American Type Culture Collection.

Bacterial Strain	Clinical Isolation	MIC (mg/mL)	MBC (mg/mL)	MBC/MIC	MBEC (mg/mL)
*S. aureus* ATCC 29213	Wound	8	16	2	24
*S. aureus* 101	Vaginal swab of a pregnant woman	8	16	2	24
*S. aureus* 104	Pharyngeal swab of a male patient	8	16	2	24
*S. aureus* 105	Urinary specimen of a male patient	8	16	2	16

**Table 7:** Evaluation of the minimum inhibitory concentration (MIC) and minimum bactericidal concentration (MBC) of hemp EO and naringenin against clinical *Helicobacter pylori* strains with different antimicrobial susceptibility patterns.

*H. Pylori* Strains	Hemp EO MIC/MBC (µg/mL)	Naringenin MIC/MBC (µg/mL)	Antimicrobial Susceptibility (µg/mL)
F4	16/16	16/16	MNZ 32; CLR > 256; AMX 0.064
E34	16/32	16/16	MNZ 1; CLR 0.064; AMX 0.016
Ro1	16/16	16/16	MNZ 1; CLR 64; AMX 0.016
Ra2	16/16	16/16	MNZ 1; CLR 128; AMX 0.016
E17	16/16	16/16	MNZ 2; CLR 256; AMX 0.064
68	16/32	16/16	MNZ 32; CLR 0.0019; AMX 0.016
ATCC 43629	8/8	16/16	MNZ 2; CLR 0.032; AMX 0.064
23	32/32	32/32	MNZ 1; CLR 0.064; AMX 0.032
Ro5	32/32	16/16	MNZ 128; CLR 16; AMX 0.125
110R	64/64	16/16	MNZ 128; CLR 0.03; AMX 0.016
F1	32/32	32/32	MNZ 2; CLR 4; AMX 0.064
190	32/32	32/32	MNZ 1; CLR 0.032; AMX 0.032
F40/499	32/32	16/16	MNZ 32; CLR 8; AMX 0.016
F40/442	32/64	8/8	MNZ 64; CLR 0.015; AMX 0.015
F34/497	64/64	16/16	MNZ 128; CLR 4; AMX 0.064

MTZ = metronidazole; CLR = clarithromycin; AMX: amoxicillin.

**Table 8:** Half maximal inhibitory concentration (IC_50_) values for the cytotoxicity of hemp EO in human cancer cells after 24 h of exposure.

Cell Line	Hemp EO	Doxorubicin
	IC_50_ (CL) μg/mL	
MCF7	83.2 (72.7–95.2)	7.6 (4.5–12.9)
MDA-MB-468	53.0 (44.2–64.3)	3.1 (2.1–4.6)
Caco-2	28.7 (17.9–45.8)	23.3 (11.1–48.3)
Mz-ChA-1	22.3 (8.3–42.5)	15.7 (6.0–31.5)
H69	nd	13.7 (7.3–25.5)

CL, confidential limits; nd, not determinable as the maximum cytotoxicity was 44.2% at the highest concentration tested (250 μg/mL). MCF-7, estrogen-dependent breast cancer cells; MDA-MB-468, triple-negative breast cancer cells; Caco-2, colorectal adenocarcinoma cells; Mz-ChA-1, cholangiocarcinoma cells; H69, nonmalignant immortalized cholangiocytes.

**Table 9:** Enzyme inhibitory effects * of the *C. sativa* samples.

Test Sample	AChE Inhibition (mg GALAE/g Extract or Oil)	BChE Inhibition (mg GALAE/g Extract or Oil)	Tyrosinase Inhibition (mg KAE/g Extract or Oil)	α-Amylase Inhibition (mmol ACAE/g Extract or Oil)	α-Glucosidase Inhibition (mmol ACAE/g Extract or Oil)	Lipase Inhibition (mg OE/g Extract or Oil)
**Hemp EO**	na	3.40 ± 0.14	35.95 ± 3.19	na	3.77 ± 0.03	70.14 ± 2.40
**Aromatic water**	2.56 ± 0.02	3.48 ± 0.02	28.24 ± 1.94	0.10 ± 0.01	0.17 ± 0.04	na

* Values expressed are the mean ± SD of three parallel measurements. GALAE: galantamine equivalent; KAE: kojic acid equivalent; ACAE: acarbose equivalent; OE: orlistat equivalent; na: not active; AChE: acetylcholinesterase; BChE butyrylcholinesterase.

## Supplementary Materials

The supplementary materials are available online.
