# Supplementary material for: Chromatographic Analyses, In Vitro Biological Activities, and Cytotoxicity of Cannabis sativa L. Essential Oil: A Multidisciplinary Study
Source: Molecules. 2018 Dec 10;23(12):3266. doi: 10.3390/molecules23123266 (PMC6320915; doi:10.3390/molecules23123266)
Supplement: Supplementary file 1 [file molecules-23-03266-s001.pdf]

Supplementary material

Chromatographic Analyses, In Vitro Biological Activities, and Cytotoxicity of *Cannabis sativa* L. Essential Oil: A Multidisciplinary Study

Gokhan Zengin [1], Luigi Menghini [2], Antonella Di Sotto [3], Romina Mancinelli [4], Francesca Sisto [5], Simone Carradori [2,*], Stefania Cesa [6], Caterina Fraschetti [6], Antonello Filippi [6], Letizia Angiolella [7], Marcello Locatelli [2], Luisa Mannina [6], Cinzia Ingallina [6], Valentina Puca [8], Marianna D'Antonio [9] and Rossella Grande [2,8]

[1] Department of Biology, Science Faculty, Selcuk University, 42130 Konya, Turkey; gokhanzengin@selcuk.edu.tr
[2] Department of Pharmacy, University "G. d'Annunzio" of Chieti-Pescara, 66100 Chieti, Italy; luigi.menghini@unich.it (L.M.); marcello.locatelli@unich.it (M.L.); rossella.grande@unich.it (R.G.)
[3] Department of Physiology and Pharmacology "V. Erspamer", Sapienza University of Rome, 00185 Rome, Italy; antonella.disotto@uniroma1.it
[4] Department of Anatomical, Histological, Forensic and Orthopedic Sciences, Sapienza University of Rome, 00185 Rome, Italy; romina.mancinelli@uniroma1.it
[5] Dipartimento di Scienze Biomediche, Chirurgiche ed Odontoiatriche, University of Milan, 20122 Milan, Italy; francesca.sisto@unimi.it
[6] Dipartimento di Chimica e Tecnologie del Farmaco, Sapienza Università di Roma, 00185 Rome, Italy; stefania.cesa@uniroma1.it (S.C.); caterina.fraschetti@uniroma1.it (C.F.); antonello.filippi@uniroma1.it (A.F.); luisa.mannina@uniroma1.it (L.M.); cinzia.ingallina@uniroma1.it (C.I.)
[7] Department of Public Health and Infectious Diseases, Sapienza University of Rome, 00161 Rome, Italy; letizia.angiolella@uniroma1.it
[8] CeSI-MeT Centro Scienze dell'Invecchiamento e Medicina Traslazionale, Center of Aging Sciences and Translational Medicine, 66100 Chieti, Italy; valentina.puca@unich.it
[9] Department of Clinical Microbiology and Virology, Spirito Santo Hospital Pescara, 65124 Pescara, Italy; dantoniomarianna2@gmail.com

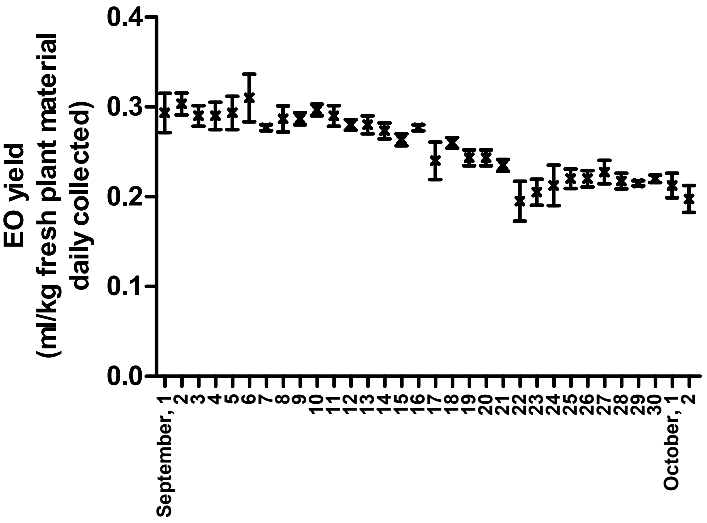

Fig. S1. Daily quantitative (mL/kg) EO yield during the flowering stage.

Table S1. Antimicrobial susceptibility pattern of *S. aureus* clinical strains by disk diffusion testing. ERY, erythromycin; TE, tetracycline; NET, netilmicin; LEV, levofloxacin; FOX, cefoxitin; LNZ, linezolid; RD, rifampicin; CN, gentamicin. S, sensitive; I, intermediate; R, resistant.

S. aureus	ERY	TE	NET	LEV	FOX	LNZ	RD	CN
ATCC 29213	S	S	S	S	S	S	S	I
101	S	S	S	S	S	S	S	S
104	R	S	I	R	R	S	S	R
105	S	S	S	S	S	S	S	S

Chromatogram for the Hemp EO (diluted 1:20 in *n*-hexane) with the relative peak of naringenin and its UV spectrum at maximum wavelength.

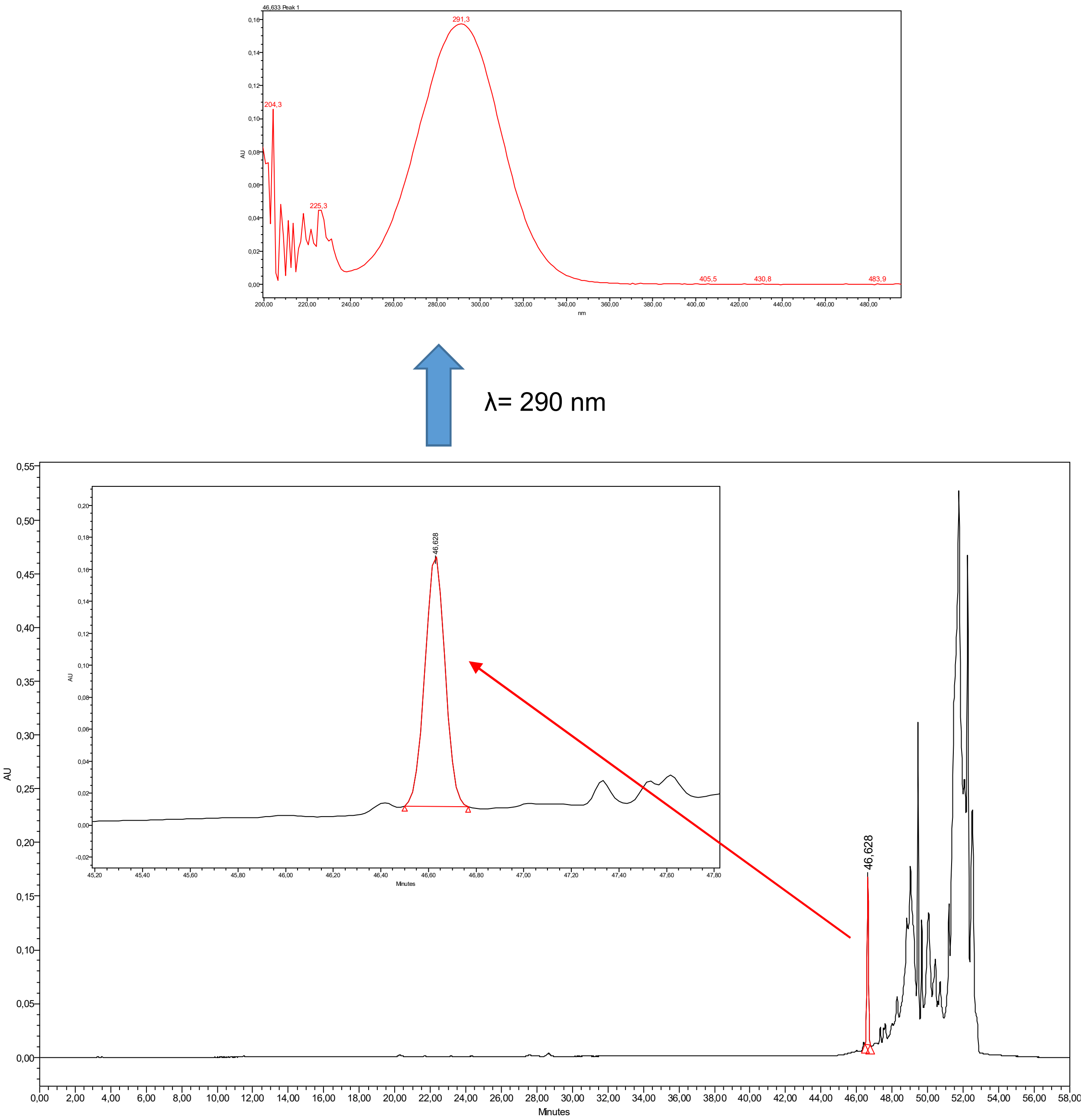

Chromatogram for the Hemp EO (diluted 1:5 in *n*-hexane) with the relative peak of naringenin and its UV spectrum at maximum wavelength.

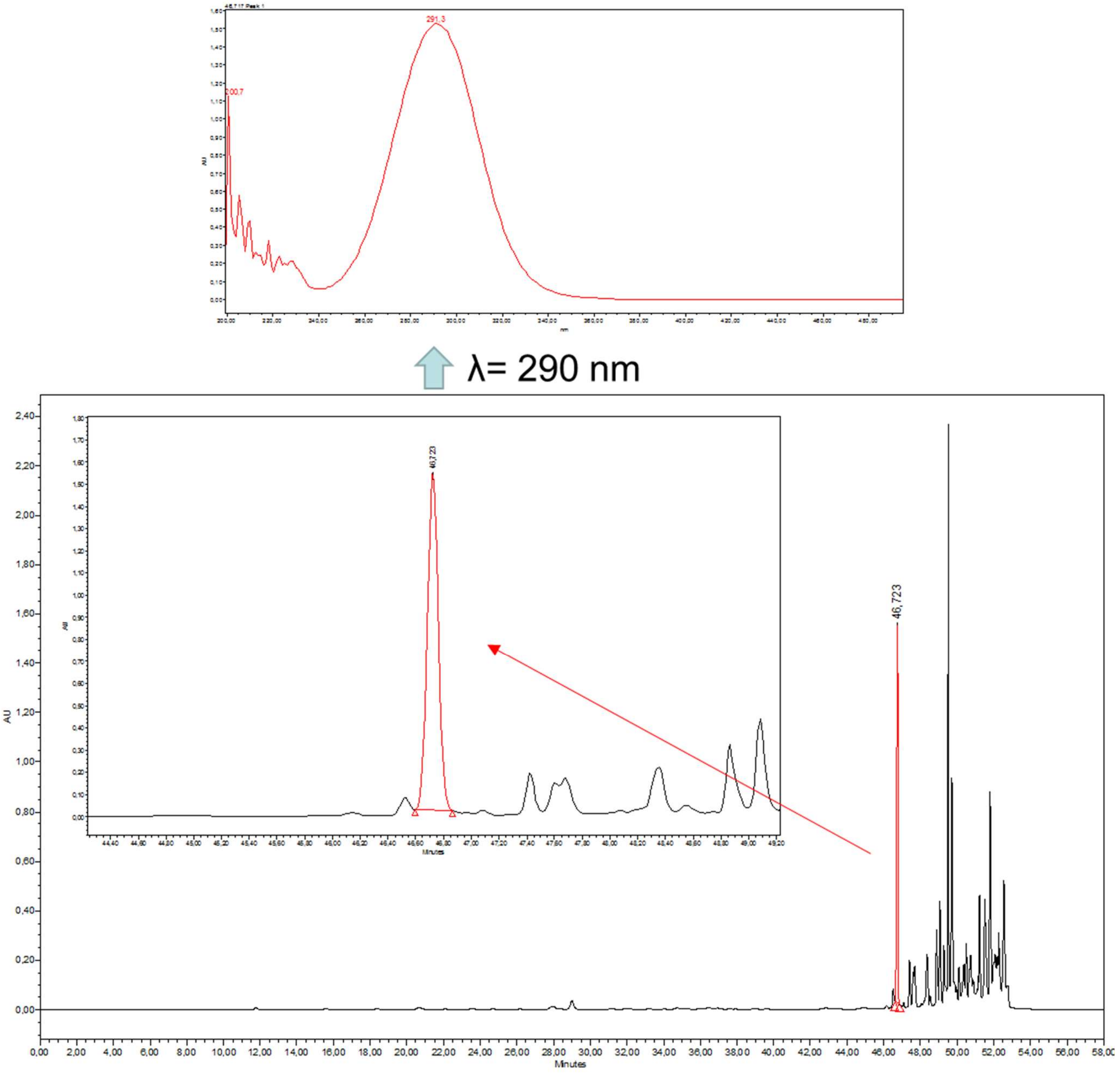

Chromatogram for the aromatic water with the relative peak of naringenin and its UV spectrum at maximum wavelength.

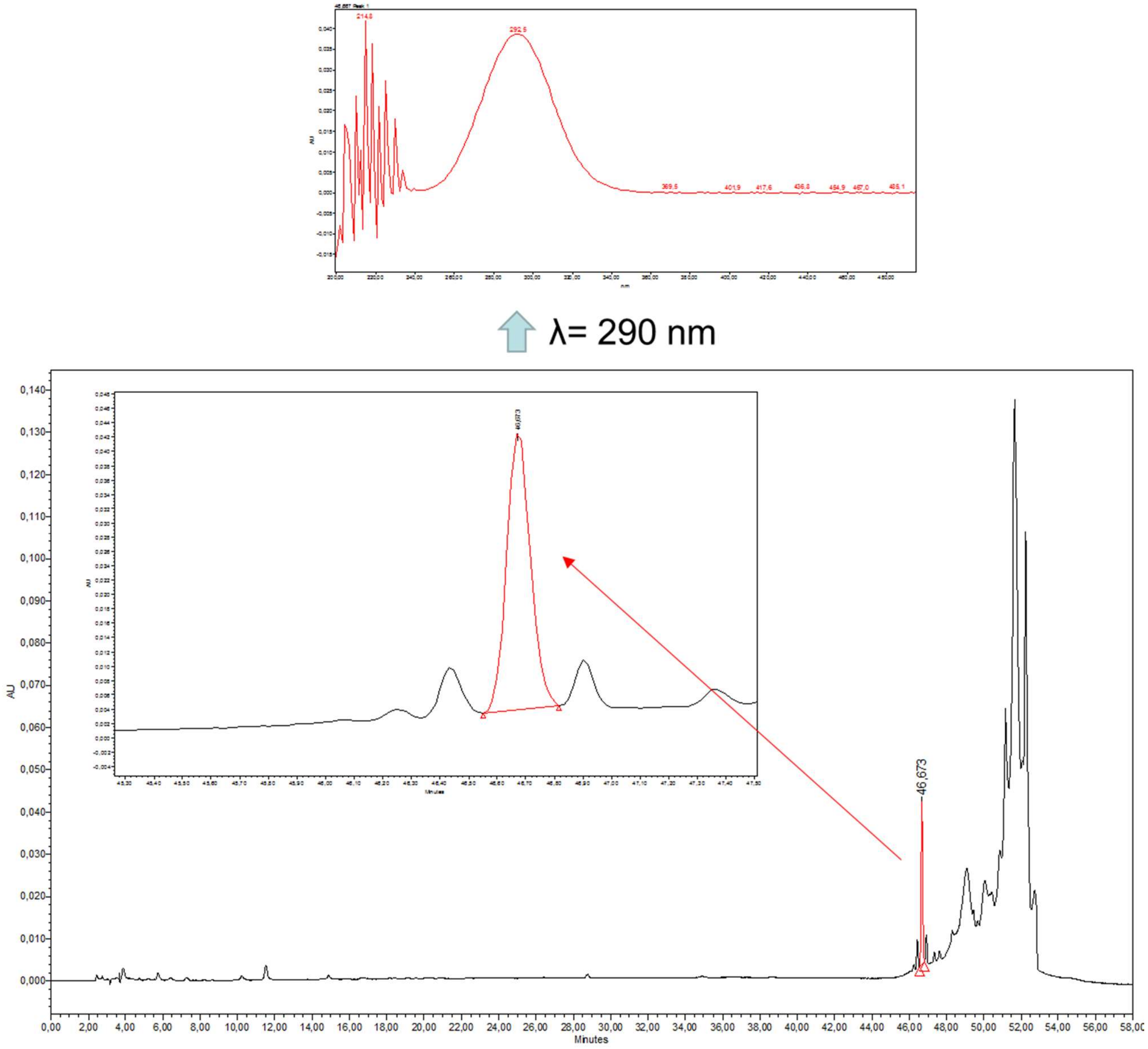
214,8
292,9
λ= 290 nm
46,673
AU
Minutes
